# Electrically Self-Healing Thermoset MWCNTs Composites Based on Diels-Alder and Hydrogen Bonds

**DOI:** 10.3390/polym11111885

**Published:** 2019-11-14

**Authors:** Guilherme Macedo R. Lima, Felipe Orozco, Francesco Picchioni, Ignacio Moreno-Villoslada, Andrea Pucci, Ranjita K. Bose, Rodrigo Araya-Hermosilla

**Affiliations:** 1 Department of Chemical Product Engineering, ENTEG, University of Groningen, Nijenborgh 4, 9747AG Groningen, The Netherlandsf.orozco.gutierrez@rug.nl (F.O.); f.picchioni@rug.nl (F.P.); 2 Laboratorio de Polímeros, Instituto de Ciencias Químicas, Facultad de Ciencias, Universidad Austral de Chile, Valdivia 5090000, Chile; imorenovilloslada@uach.cl; 3 Department of Chemistry and Industrial Chemistry, University of Pisa, Via Moruzzi 13, 56124 Pisa, Italy; andrea.pucci@unipi.it; 4 Programa Institucional de Fomento a la Investigación, Desarrollo e Innovación, Universidad Tecnológica Metropolitana, Ignacio Valdivieso 2409, P.O. Box 8940577, San Joaquín, Santiago 8940000, Chile

**Keywords:** Paal-Knorr reaction, polyketone, carbon nanotubes, nanocomposite, Diels-Alder, click-chemistry, hydrogen bonding, self-healing, re-workability, recycling, Joule heating

## Abstract

In this work, we prepared electrically conductive self-healing nanocomposites. The material consists of multi-walled carbon nanotubes (MWCNT) that are dispersed into thermally reversible crosslinked polyketones. The reversible nature is based on both covalent (Diels-Alder) and non-covalent (hydrogen bonding) interactions. The design allowed for us to tune the thermomechanical properties of the system by changing the fractions of filler, and diene-dienophile and hydroxyl groups. The nanocomposites show up to 1 × 10^4^ S/m electrical conductivity, reaching temperatures between 120 and 150 °C under 20–50 V. The self-healing effect, induced by electricity was qualitatively demonstrated as microcracks were repaired. As pointed out by electron microscopy, samples that were already healed by electricity showed a better dispersion of MWCNT within the polymer. These features point toward prolonging the service life of polymer nanocomposites, improving the product performance, making it effectively stronger and more reliable.

## 1. Introduction

Self-healing thermoset polymer materials represent an outstanding approach and cost-effective solution for non-recyclable thermoset applications [1]. These materials possess the remarkable ability of being mended, which thus prolongs their service life [2]. Particularly, the self-healing approach in thermoset polymer materials relies on the ability of the linking moieties to cleave—and—reform upon exposure to a certain stimulus. Thus, microcracks on the material can be repaired based on such reversible features [3,4]. For instance, such a healing effect can be triggered by heat [5,6,7,8] or light [9,10,11,12,13].

Intensive research efforts are also dedicated to the synthesis of self-healing thermoset polymers by including organic/inorganic nanofillers during in-situ polymerization to improve the nanofiller distribution, strength, modulus, and toughness of the final thermoset nanocomposite material to achieve further improvements [14,15,16,17]. As a normal procedure, the weight content of the nanofiller (e.g., carbon nanotubes) in the matrix is tailored to gain the maximal reinforcement from the filler while considering its aspect ratio and loading. Accordingly, the chemical functionalization of nanofillers (via covalent attachment of compatibilizing functional groups or polymers) has been reported to increase the homogeneous distribution of the filler and the mechanical performance of the nanocomposites, even while using relatively high filler loadings [16,18,19,20]. Although nanocomposites with exceptional mechanical and self-healing properties have already been produced [4,21,22], the healing process for most of the applications still relies on direct heating as external stimulus for repairing. The latter hinders macro-scale applications of these materials due to the high-energy source used for in-situ (i.e., in-service) damage repairing [23].

Recently, many authors have reported approaches for in-situ damage repairing with remarkable achievements [24,25,26,27]. Among those, a newly emerging system is represented by electrically self-healing nanocomposite. Electrically-induced self-healing is a relatively new concept that is currently used in the design of light long-lasting nanocomposites for electronic applications and actuators [28,29,30,31,32,33]. The healing process occurs via heat generation when an electrical current passes through a conductive matrix, such as a percolated multi-walled carbon nanotubes (MWCNT) network. The so-called Joule-effect activates the intrinsic self-healing ability of thermally self-mendable matrices to heal damage in local areas [34,35]. Among the several chemical routes used for thermal healing, the Diels–Alder (DA) reversible cycloaddition is one of the most effective alternatives [36]. It allows for the formation of three-dimensional and reversible network structures by means of the DA and retro-Diels-Alder (r-DA) reactions. Besides, thermally-reversible polymers based on DA chemistry and combined with MWCNTs have proven to be feasible systems for generating electrically self-healing nanocomposites based on the DA reaction [37]. In this particular example, the activation of the Joule heating, using electricity in the presence of macroscale damages (i.e., cuts), generates local changes in electrical resistivity at the crack tip, which leads to a local temperature increase, within the range of the r-DA reaction temperatures. The elevated temperature generates sufficient chain mobility to close and seal the cut upon cooling by means of the DA reaction. Remarkably, this approach introduced a new concept for polymer healing while using Joule heating and set the basis for the development of self-healing electrically conductive nanocomposites. It is, in fact, a fundamental point, since the higher interfacial interaction between the components via DA chemistry, the better the recovery of mechanical properties. For instance, it has been already reported that MWCNTs promotes a catalytic effect upon DA reaction for damage recovery in thermally self-healing matrices that are based on reversible DA chemistry [38].

Owing to the need to produce scalable self-healing thermoset nanocomposite systems, the chemical modification of polyketones (PK) with amine compounds via the Paal–Knorr reaction represents a feasible alternative for the industrial production of these materials [39]. This is related to the tolerance of this reaction towards many functional groups, particularly sterically hindered amines. This synthetic pathway offers several advantages, such as high yield under relatively mild conditions, high reaction kinetics, even in the absence of any catalyst, and water as the only by-product. In 2009, Picchioni and his co-workers published the first attempt for the chemical modification of PK with furan amino-substituted compounds aimed at preparing thermoset polymer networks that are able to undergo reversible reactions via DA chemistry [40]. The furan groups that were directly grafted on the PK backbone chain allowed for the formation of a three-dimensional network structure after being cross-linked with aromatic bismaleimide (B-Ma). The material indeed formed a thermally reversible and self-healing thermoset polymer network by means of the DA and r-DA sequence. In another approach, polyketones bearing DA and hydrogen bonding (HB) active groups were prepared by the same Paal–Knorr chemical route. The resulting materials displayed self-healing properties while using heat as external stimulus [41]. The novelty of this approach allowed for the preparation of a series of compounds displaying the same backbone structure but a systematic variation in the amount of hydrogen bonding and Diels–Alder pendant groups. This allowed for precisely pinpointing the influence of both kinds of interactions on the thermal and mechanical properties and thermal self-healing of the systems.

In a previously reported work, we have demonstrated the electrical-conductivity of a composite material consisting of a polyketone functionalized with furan groups, cross-linked with bismaleimide, and reinforced with MWCNTs [42]. Herein, we report on electrically self-healing nanocomposites that display tunable thermomechanical properties and re-workability by means of tuning the ratio between DA and HB functional groups and the wt.% loading of MWCNTs. These electrically conductive nanocomposites stem from the chemical modification of an alternating aliphatic polyketone grafted with furan (PK-Fu) and propyl alcohol groups (PK-Fu-A2P) via the Paal–Knorr reaction. The polymers are cross-linked with B-Ma and reinforced with MWCNTs via reversible Diels–Alder cycloaddition and hydrogen bonding. Spectroscopic techniques were used to study the chemical modification of the polyketone backbone polymer and crosslinking of the resulting polymer nanocomposites via DA reaction. Thermomechanical tests allowed for evaluating the mechanical performance, recyclability, and re-workability of the composites via reversible DA/r-DA cycloaddition, hydrogen bonding, and MWCNTs loading. The modulus and electrical performance of the material were measured. Additionally, the effect of Joule heating was followed by IR thermography. The morphology of the systems was studied by optical microscopy. The dispersion of MWCNTs in the polymeric matrices was analyzed by electronic microscopy before and after self-healing by Joule heating.

## 2. Materials and Methods

The alternating aliphatic polyketone (PK30) was synthesized according to previously reported works [39,40,41]. The resulting co- and terpolymers of carbon monoxide present a total olefin content of 30% of ethylene and 70% of propylene (PK30, MW 2687 Da). Furfurylamine (FU, Sigma-Aldrich, ≥99%, Zwijndrecht, The Netherlands), and amino-2-propanol (A2P, Sigma Aldrich, 99%, Darmstadt, Germany) were freshly distilled before used. Multi-walled carbon nanotubes (MWCNTs, O.D. 6–9 nm, average length 5 µm, Sigma-Aldrich 95% carbon, St. Louis, MO, USA), dimethyl sulfoxide-d6 (DMSO-d6, Sigma-Aldrich 99.5%, St. Louis, MO, USA), (1,1-(methylenedi-4,1-phenylene)bismaleimide (B-Ma, Sigma-Aldrich 95%, St. Louis, MO, USA), tetrahydrofuran (THF, Boom BV ≥95%, Meppel, The Netherlands), chloroform (CHCl_3_, Avantor 99.5%, Gliwice, Poland), and deuterated chloroform (CDCl_3_, Sigma-Aldrich 99.8 atom% D, St. Louis, MO, USA) were purchased and used as received.

### 2.1. Functionalization of Polyketone with Furan and Propyl Alcohol Groups

**Figure 1:**
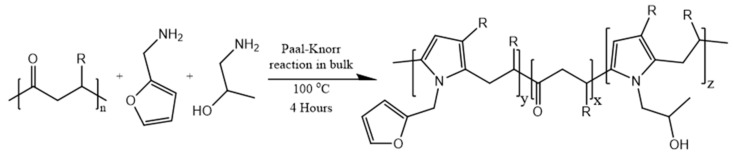
Schematic representation of Paal–Knorr functionalization of Polyketone with furfurylamine and amino-2-propanol (PK-FU-A2P).

The reaction between PK30, FU, and A2P (Figure 1) was carried out as described by Araya-Hermosilla et al. [41] at different ratios between the 1,4-dicarbonyl groups of PK30 and the primary amine groups of FU and A2P via the Paal–Knorr reaction. The molar ratio between the reactants in the feed and methodology are described in the Table S1. The chemical modifications of PK30 yielded different polymers bearing pendant furan and hydroxyl groups (PK30_x_FU_y_A2P_z_).

### 2.2. Preparation of PK30_x_FU_y_A2P_z_/B-Ma/MWCNT Composite

A three-dimensional and reversible network structure was produced by means of the DA reaction of the furan-derived PK30 and the aromatic bismaleimide cross-linker agent. MWCNTs may also undergo DA with furan and maleimide groups by means of the diene/dienophile character of the graphitic surface of the MWCNTs [43]. First, the MWCNTs were suspended in CHCl_3_ (0.75 wt.%), a solvent reported as an effective dispersant for MWCNTs [44,45], and sonicated for 30 min. Polymers that were grafted with FU and A2P groups at different ratios, bismaleimide at equimolar amounts with the furan groups, and already sonicated MWCNTs at specific percentages from 0.1 to 5 wt.% (Table S2) were mixed in chloroform (comprised roughly 90% of the total volume) in a round-bottom flask with a condenser. The reaction was set under vigorous stirring at 50 °C for 24 h while using an oil bath that was equipped with a temperature controller. The resulting mixtures were put on Teflon plates to evaporate the solvent in a vacuum oven at 60 °C for 48 h. Around 500 mg of the powder acquired from ground materials after DA reaction was set in stainless steel molds that were lined with Teflon paper. The powders were then pressed using a heated press (Schwabentan Polystat 100T) at 150 °C and 40 bar pressure for 30 min. to form rectangular bars with dimensions of 35 mm × 6 mm × 1 mm. The bars were cooled down to room temperature and stored for further testing.

### 2.3. Characterization

The elemental composition of the polymers was analyzed while using a Euro EA elemental analyzer (Langenselbold, Germany) for nitrogen, carbon, and hydrogen. ^1^H-NMR spectra were recorded on a Varian Mercury Plus 400 MHz apparatus (Agilent, Santa Clara, CA, USA) while using CDCl_3_ or DMSO-d6 as solvent. FT-IR spectra were collected using a Perkin-Elmer Spectrum 2000 (San Francisco, CA, USA), transmission measurements were recorded at the range of 4000 cm^−1^ to 500 cm^−1^ at a resolution of 4 cm^−1^ averaged over 64 scans. Differential scanning calorimetry (DSC) analysis was performed on a Perkin Elmer Pyris Diamond under a nitrogen atmosphere (Shelton, CT, USA). The samples were weighed (5–12 mg) in an aluminum pan, which was then sealed. Subsequently, the samples were heated from 0 °C to 150 °C and then cooled to 0 °C. Four heating-cooling cycles were performed at a rate of 10 °C/min. Gel Permeation Chromatography (GPC) measurements were performed with an HP1100 Hewlett-Packard (Wilmington, Philadelphia, PA, USA). The equipment consists of three 300 × 7.5 mm PLgel 3 m MIXED-E columns in series and a GBC LC 1240 RI detector (Dandenong, Victoria, Australia). The samples were dissolved in THF (1 mg/mL) and eluted at a flow rate of 1 mL/min. and a pressure of 100–140 bar. The calibration curve was made using polystyrene as standard and the data were interpolated using the PSS WinGPC software. Thermomechanical analyses were conducted on a Perkin Elmer Dynamic Mechanical Analyzer DMA 8000 (Waltham, MA, USA) using single cantilever mode at an oscillation frequency of 1 Hz and heating rate of 3 °C/min. The samples for DMA analysis were prepared by compression molding of 500 mg of the composite into rectangular bars (6 mm wide, 1 mm thick, 35 mm long) at 150 °C for 30 min. under a pressure of 40 bar to ensure full homogeneity and then annealed in an oven at 50 °C for 24 h. Electrical measurements were performed on the rectangular bars used for DMA analysis. The setup consisted of a Velleman Power supply single output DC switching bench 60V, 5A, and a multimeter (Gossen Metrawatt Metrahit 18S) (Figure S1). Electrical parameters were measured on samples that were connected to a conventional circuit using copper clamps holders. In addition, silver paste was used at both ends of the bars and cover into aluminum foil to improve the contact area between the copper clamps holder and the sample. The sample resistivity (ρ) was calculated, as follows

(1) $$\rho =R\frac{A}{l}$$

where ρ is given in Ω·m, **R** (Ω) is the electrical resistance, **A** (m^2^) is the cross-sectional area, and **l** (m) is the length between copper clamps holders. The electrical resistance is calculated, as follows

(2) $$R=\frac{V}{I}$$

where **V** stands for voltage (V) supplied in the electrical circuit and **I** is the current (A) measured in amperes passing through the sample. Thermal images of the samples that were subjected to electrical current were obtained while using a Fluke IR thermometer camera (VT02 Everett, WA, USA) (Figure S1).

For safety and practical reasons, when the temperature of the different samples reached 150 °C, the stimulus that triggered the rise in temperature was switched off, such as heat exchange in DSC and DMA, and voltage in conductivity analyses.

## 3. Results and Discussion

### 3.1. PK Functionalized with Furan and Propyl Alcohol Groups Via Paal-Knorr Reaction

The Paal–Knorr reaction on the polyketone with FU and A2P resulted in dicarbonyl conversion of higher than 65%, as seen in Table 1. This is a good result, given that the maximum conversion expected has been demonstrated to be statistically 80% [41], due to the occurrence of reactions leaving the monocarbonyl segments in the chains. The total conversion efficiency (*y* + *z*) could be calculated by EA through the relative content of *N* in the samples. The relative weight of *y* and *z* could be obtained by ^1^H-NMR.

**Figure 2:**
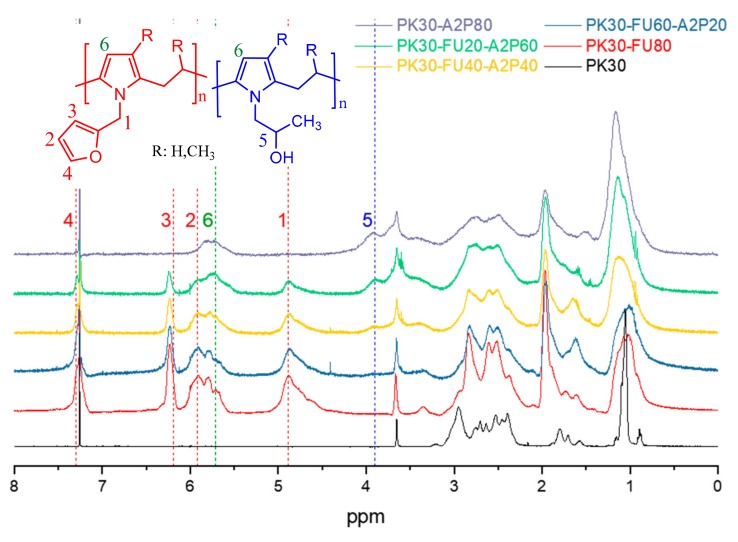
^1^H-NMR spectra of PK30 functionalized FU and A2P at different ratios.

Figure 2 shows the ^1^H-NMR spectra of the polymers before and after the different functionalizations. In all cases, a successful grafting process was indicated by the signals that were attributed to the formed pyrrole rings around 5.7 ppm (H6). Peaks at 4.9 (H1), 5.9 (H2), 6.2 (H3), and 7.3 ppm (H4) confirm the presence of furan groups, while 3.9 ppm (H5) signal stands for the confirmation for the hydroxyl moieties. The spectra clearly show how the signals attributed to each group increase or decrease with the respective FU/A2P ratio used in the formulation. From the relative intensity of the resonance peaks, the values of *x* and *y* shown in Table 1 were obtained.

**Figure 3:**
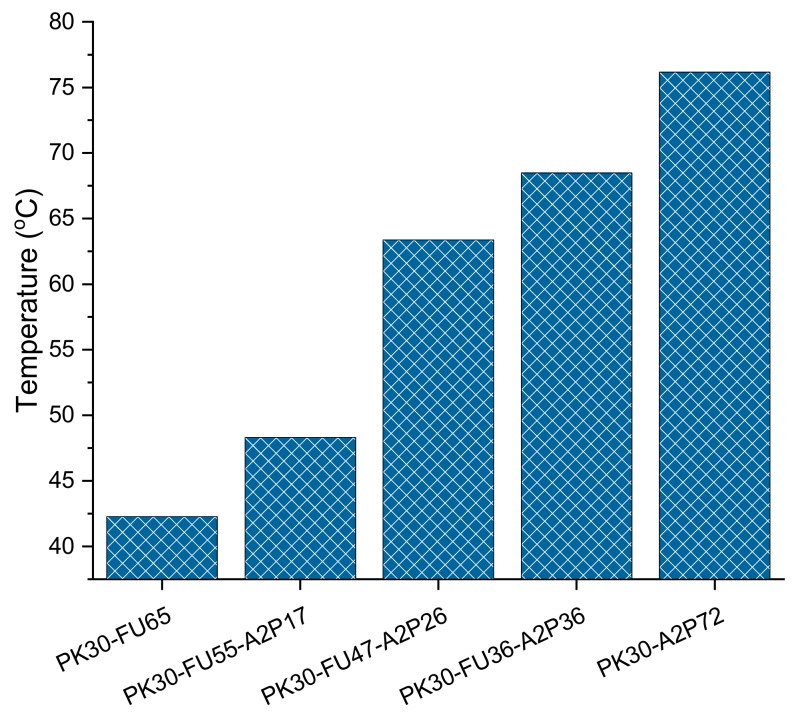
Glass transition of PK30 grafted with FU, A2P, and their respective ratios according to DSC (see Figure S2 for thermal history of all polymer series).

DSC experiments of the polymers show differences in their respective T_g_, as can be seen in Figure 3. The experimental plots can be seen in Figure S2, where the first curves were neglected to remove the thermal history. As expected, polymers with stronger intermolecular HB showed higher T_g_. The T_g_ increased as a function of the A2P/FU ratio for all of the different copolymers synthesized.

### 3.2. PK-Fu-A2P / B-Ma/ MWCNT Composite

**Figure 4:**
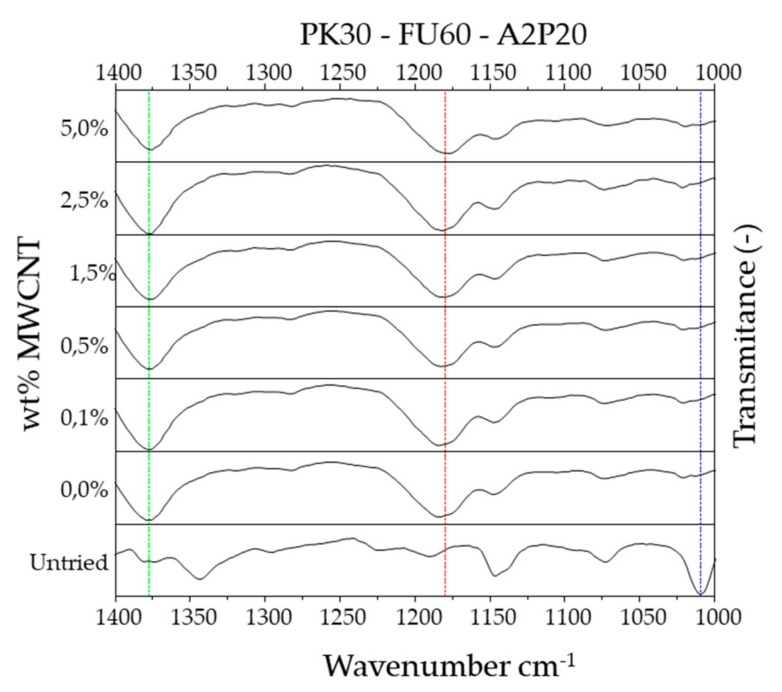
FT-IR of polyketones (PK) grafted with the ratios of FU and A2P Untried (not cross-linked) and cross-linked with different wt.% of multi-walled carbon nanotubes (MWCNTs).

The polymers were mixed with bismaleimide and MWCNT while using CHCl_3_ as solvent at 50 °C for 24 h to produce the reinforced composite materials through the DA cycloaddition reaction. Figure 4 and Figure S3 show the FT-IR spectra of the composites before and after the cross-linking reaction in the absence and presence of 5 wt.% MWCNT [43,46,47]. There are emerging bands centred at approximately 1180 cm^−1^ attributed to the C-O-C ether moiety from the DA adduct. The band at 1009 cm^−1^ assigned to C-O-C of unreacted furans, and a band at approximately 1378 cm^−1^, representing C-N stretching in the maleimide ring disappear over time.

**Figure 5:**
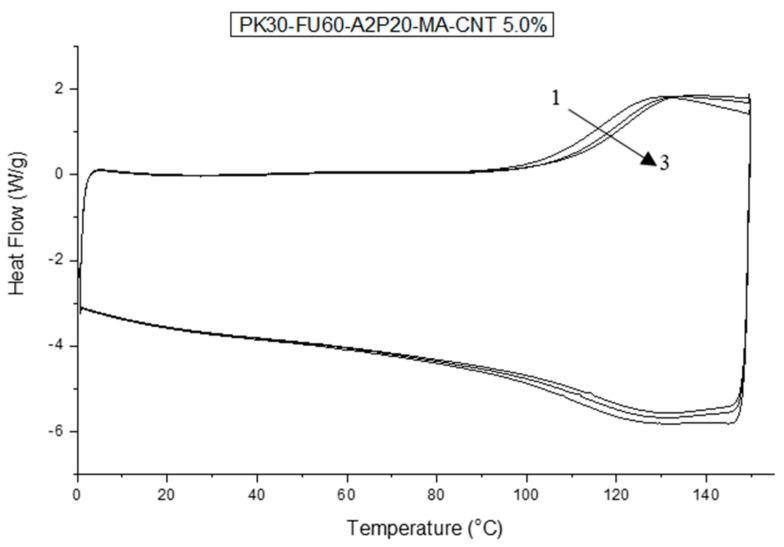
Differential scanning calorimetry (DSC) thermal cycles of PK-Fu60-A2P20 cross-linked with b-Ma and reinforced with 5.0 wt.% of MWCNTs.

The DSC experiments were then performed with the cross-linked nanocomposites to follow the reversibility process related to the DA and r-DA sequence of the materials. For brevity, only one DSC study is shown in Figure 5 (see Figure S4 for a full review of all crosslinking series of nanocomposites). The resulting graph displays a broad endothermic transition in the range of temperature 100–140 °C for each consecutive thermal cycle. The resemblance between the cycle curves in the DSC graphs confirm the reversible cross-linked character of the samples, in other words, the resilience of the material upon thermal cycling.

**Figure 6:**
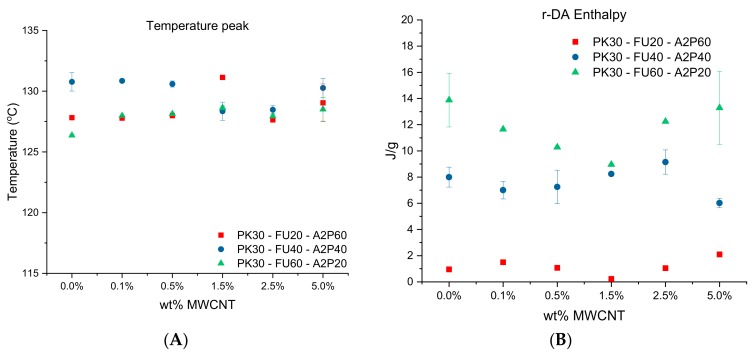
(**A**) Temperature peak where most of the Diels–Alder (DA) adducts are cleaved for all the series of cross-linked nanocomposites with bismaleimide and different wt.% of MWCNTs. (**B**) Enthalpy as determined by DSC analysis of cross-linked samples according to wt.% of MWCNT and ratios of amines compounds.

In addition, from DSC analysis it was possible to evaluate the de-cross-linked samples. We can assign many parameters to compare the samples since the endothermic transition corresponds to the r-DA process. Thus, the peak of the curves in Figure 5 is the indication of the moment at which the DA cleaved is at its maximum. The area under the curve associated to this peak is therefore related to the energy that is absorbed during the cleavage of the DA adducts [48]. That is to say, the results (Figure 6A) present no relevant changes or trends relating temperature peaks to the ratio of amines or the weight percentage of MWCNT in each sample. This result can be interpreted as the intermolecular bonds playing an important role in the system. Hence, a greater amount of FU results in a higher crosslinking density. The latter means that, even though the DA cross-linking density diminished, physical interactions via hydrogen bonding can compensate the thermal stability of the systems. This is supported by the DSC results shown in Figure 3, where the higher amount of OH groups results in higher glass transition temperatures of the different polymers [8,42].

Likewise, the area under the curve, also being addressed as the endothermic integral, does not present the trend of relation in the manner of different weight percentage of MWCNT (Figure 6B). Nonetheless, there is a pertinent difference of area if we take the different amine ratios into consideration, and clearly, the percentage of FU that is grafted onto the polymer dictates the energy absorbed during the cleavage of the DA adducts. This is in agreement with the cross-linking density of the material as already mentioned [46]. 

### 3.3. Mechanical Properties

**Figure 7:**
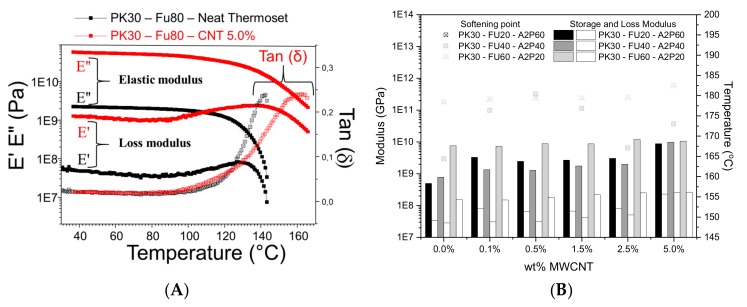
(**A**) Dynamic mechanical analysis of PK30-FU80 (reference sample) cross-linked with bismaleimide and reinforced with 5 wt.% of MWCNTs. Black line represents the sample without filler and the red one crosslinked with 5 wt.% of MWCNT (**B**) E’ E”(bars–left axis), tan (δ) delta (points–right axis) vs Temperature for formulations that vary on the Fu/A2P ratio and different wt.% of MWCNTs (see Figure S6 for the whole series of DMA analysis).

Successful bar specimens for DMA analysis were obtained after hot compression molding of ground cross-linked samples using 150 °C and 40 bar during 30 min. This process favors the r-DA mechanism that leads to the de-crosslinking of the thermoset nanocomposites (Figure S5 grinded/molded samples). This probes the reversibility and recyclability character of these materials. The variation in storage modulus (E’-ability of the material to store energy), loss modulus (E”-ability of the material to dissipate energy), and ratio of the loss modulus to the storage modulus (tan δ) of all the samples were measured while using the single cantilever method in the DMA analyses. The peak of tan δ is where the viscous behavior of the network is at its maximum. For simplicity, we refer to this as the softening point in this discussion. Figure 7A shows that the filler considerably enhances the loss/elastic moduli and softening point (tan(δ) peak), with respect to the neat cross-linked thermoset.

Remarkably, the storage and loss moduli increased more than one order of magnitude when comparing the neat thermoset system to the reinforced one containing 5 wt.% of MWCNTs (Figure 7A). Likewise, the softening point (tan(δ)) increased from ~140 °C (neat thermoset) to ~160 °C for the reinforced nanocomposite. This can be explained by the MWCNTs reinforced structure of the thermoset matrix showing increased rigidity. This effect might also be a consequence of strong interfacial interactions between the polymer matrix and MWCNTs. As furan and maleimide moieties both have been reported to undergo DA cycloaddition with CNT surface [38,42]. In Figure 7B, the ratio of DA and HB active groups grafted on the neat thermosets (without MWCNTs) shows increasing E’, E”, and tan δ with increasing crosslinking density. However, substantial differences are not found in E’, E” and tan δ between systems with the same Fu/A2P ratio in the presence of increasing wt.% of MWCNTs. This suggests the lack of interaction between the nanofiller and the cross-linked thermoset matrices. We also observed that systems with different Fu/A2P ratios and same 5 wt.% of MWCNTs show similar E’, E”, but the tan(δ) is 10 °C higher for the highest cross-linked PK30–Fu60–A2P20 system. As compared to the PK30–Fu80 reference system with 5 wt.% MWCNTs, the nanocomposites PK30–Fu60–A2P20 showed a 22 °C higher softening point (tan(δ)). This reveals the greater capacity for dissipating energy most likely due to the better distribution of MWCNTs and the contribution of HB in the matrix.

### 3.4. Electrical Conductivity Properties

The electrical conductivity measurements were performed to evaluate the minimum amount of MWCNTs needed to achieve an efficient percolation pathway to heat the sample by Joule heating. The sample bar was placed between two copper clamps (Figure S1). Silver paste and aluminum foil were used to improve the contact between the copper clamps and the samples. An infrared camera is used in addition to a digital thermometer with a thermocouple to monitor the temperature change during the conductivity tests (Figure S1).

**Figure 8:**
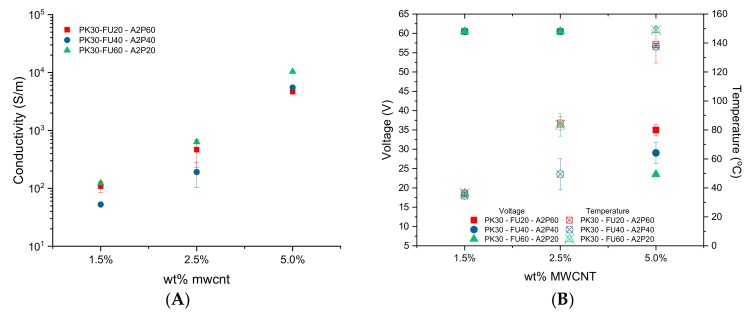
(**A**) Electrical conductivity of sample bars and (**B**) Temperature reached with each sample bars according to voltage.

The minimum amount of MWCNTs needed to obtain electrical conductivity is 1.5 wt.% for all three different ratios between Fu/A2P functional groups, as can be seen in Figure 8A. Conductivity increases as the amount of MWCNTs increases by two orders of magnitude higher for either composite system containing 5 wt.% MWCNTs, as compared to systems only containing 1.5 wt.%. The systems containing FU60/A2P20 ratio conduct more electricity than systems containing lower FU/A2P ratios, even though the amount of MWCNTs is the same in all instances. This characteristic has been already been reported previously and attributed to the effective primary (covalent) and secondary interactions between MWCNT and FU. Both types of interactions cooperatively favor the homogeneous distribution of the filler along the sample [42,49].

Importantly, the temperature that the bars achieve by means of the Joule heating under different voltages was measured with the same set up using the thermal camera. Temperatures above 120 °C are needed to activate the r-DA process for self-healing with samples containing 5 wt.% of MWCNTs. This required using voltages between 25 to 35 V (Figure 8B). For lower MWCNTs content, the temperature did not reach such a high value, even upon applying voltage of 60 V. This is clearly related to the ratio between Fu/A2P compounds, the higher amount of FU grafted, the higher the achieved temperature with lower voltage.

Notably, the conductivity and Joule heating are reproducible during heating and cooling cycles (e.g., turning on/off the circuit), thus testifying that the percolation pathway does not undergo relevant alteration in the course of temperature changes. Moreover, according to the conductivity tests, the higher the FU content in the sample, the greater was the overall bar conductivity, which resulted in improved resistive heating being needed to perform electrical self-healing. That is to say that the MWCNTs distribution was superior in the sample PK30-FU60-A2P20 than in other FU/A2P ratios.

### 3.5. Thermal and Electrical Self-Healing

**Figure 9:**
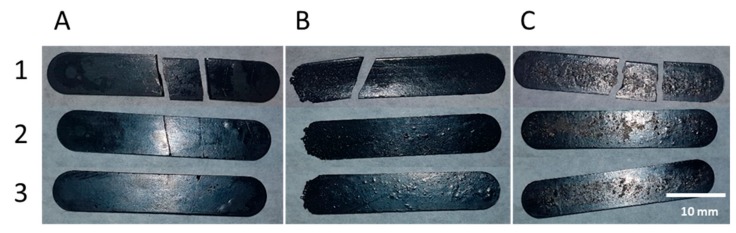
Thermally self-healing of sample during the re-process of the bars with press using 150 °C and 40 bar for 30 min. Sample (**A**) PK30-FU20-A2P60-CNT 0.0% (**B**) PK30-FU20-A2P60-CNT 1.5% (**C**) PK30-FU60-A2P20-CNT 0.1% at the moment (1) broken, (2) healed front face (3) healed back face.

Two qualitative experiments were done in order to demonstrate the self-healing features of the nanocomposites. The first test attempted to validate the self-healing process by breaking bar samples. For all the wt.% of MWCNT, the broken samples were placed back in the molds where they were first made. The initial process conditions of 150 °C and 40 bar for 30 min. were applied. The bars recover their full integrity, as can be seen in Figure 9. The effectiveness of this method is expected, since applied pressure enhances the contact between the damaged faces after the r-DA has taken place, allowing for the DA reaction to proceed upon cooling. This method not only proves self-healing ability, but also the reworkability and recyclability of the material.

**Figure 10:**
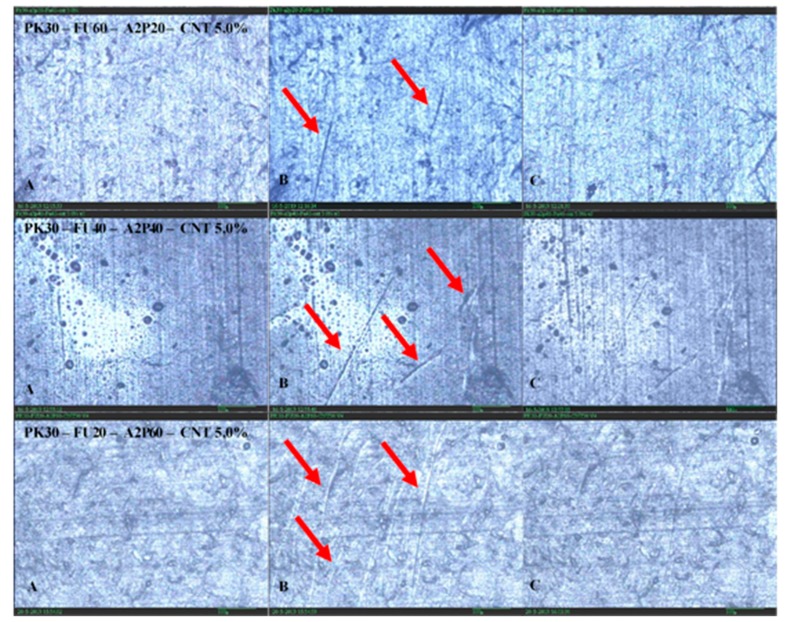
Microscope images of polyketone grafted with different ratio of amines (Fu/A2P) and cross-linked during the healing process. Same area with (**A**) no damage, (**B**) damaged, and (**C**) healed.

In the second test, samples with 5.0% MWCNTs were set in the same frame used to evaluate the conductivity of the bars, under a microscope. Figure 10A,B show the samples before and after damaging, respectively. The scratches on the surface of the samples were done by hand with a sharp blade. Subsequently, an electrical current is applied to produce the Joule heating in the materials under a voltage ranging from 25 to 35 V. After the sample reached a temperature of about 145 °C, healing was verified, as can be seen in Figure 10C, showing nearly full recovery from the damage. It is important to mention that all of the samples with different ratios of amines successfully healed after 10 min.

**Figure 11:**
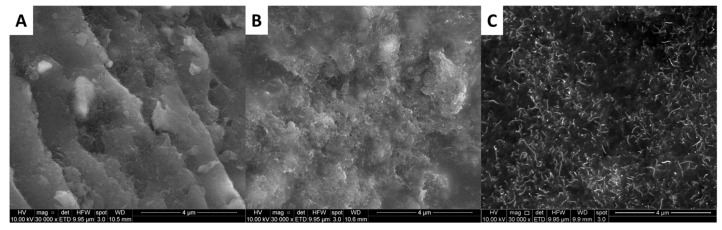
SEM micrographs of PK30-FU60-A2P20-CNT 5% cross-linked composite after molding (**A**), heating in an oven at 150 °C for 24 h (**B**) and after Joule heating at 145 °C using 25 Volt during 24h (**C**).

The SEM micrographs were obtained from the bar sample PK30-FU60-A2P20-CNT 5% subjected to different treatments. Figure 11A shows the freshly broken surfaces of the composite after molding, where delamination and poor dispersion of the MWCNTs were observed. Figure 11B displays the same sample after being molded and annealed by conventional heating at 150 °C for 24 h. It is worth noticing that a better MWCNTs dispersion was achieved and delamination was diminished, suggesting the better interaction between the components [42]. Notably, Figure 11C showed that the sample subjected to electrical current rendered a composite with well dispersed MWCNTs. As compared to Figure 11A,B, the Joule heating increased the polymer mobility that favors the redistribution of macromolecular chains, thus increasing the MWCNT dispersion aided by the better interfacial adhesion between the components.

## 4. Conclusions

In this project, we have demonstrated the design and development of thermoset polymer nanocomposites showing tunable mechanical, electrical, and self-healing properties. The materials are capable of recovering structural damage due to thermally reversible DA bonds that are assisted by HB through Joule heating. The effect the amine content and the fraction of the fillers on the self-healing, electrical and mechanical properties of the final products were systematically investigated. The self-healing nanocomposite materials were prepared via the Paal–Knorr modifications of an alternating aliphatic PK with the association of DA reversible reaction (Furfurylamine) and HB (Amino-2-propanol) to assist the healing process. In addition, MWCNT was included to provide reinforcement as well as an electrical pathway. The reactions were successfully carried out in a one-pot method and FT-IR, ^1^H-NMR analysis, elemental analysis, and GPC verified its high-efficiency yield. The thermal properties of the polymer evaluated by DSC before crosslinking showed the relevant contribution of HB groups to the glass transition.

Likewise, FT-IR analysis confirmed the presence of the DA adduct proving successful cross-linking reaction. DSC confirmed the thermal reversibility of the nanocomposites where consecutive cycles approximately overlap each other in all of the samples. The electrical measurements showed the correlation of weight content of MWCNT and resistivity. It proved that a moderate amount of MWCNT filler, more precisely 1.5 wt.%, was enough to build an electrical network inside the nanocomposite; however, this amount of filler was not enough to heat the sample with a voltage of 60V. However, samples with 5.0 wt.% of MWCNT could reach temperatures high enough to undergo r-DA reaction. Interestingly, the electrical measurements also established that samples with a higher ratio of FU conduct electricity better if compared to lower ratios due to the better interfacial adhesion between the components. This suggests the ability of FU groups to react with MWCNTs and possibly assist the distribution of MWCNT during the dispersion. With regard to self-healing and reworkability, the nanocomposite presented excellent results for all of the methods applied. The conventional way in the oven and the resistive heating could both achieve almost a complete disappearance of the damage in the samples.

This research might contribute towards a cost-effective industrial production of electrically-conductive, recyclable thermoset nanocomposites featuring re-connection that is triggered by Joule heating. As compared to published works on electrically self-healing systems, the present materials are crosslinked and recyclable nanocomposite systems that can be used as an additive or matrix for the manufacture of materials. Particularly, alternating aliphatic polyketones containing furan pendant groups act as diene polymer materials that are of interest to academia and are industrially scalable. In line with that, the article also shows a robust method for obtaining nanocomposites that can be used as thermosetting, thermo-reversible, thermo-adhesive, thermo-conductive, electrically-conductive, and self-repairing systems.

## Figures and Tables

**Table 1:** Elemental Analysis and Gel Permeation Chromatography (GPC) measurements of PK30 alone and modified with Furfurylamine (FU) and A2P at different ratio.

Sample	x (%)	y (%)	z (%)	CO (%)	Mn (×10^3^ M)	Mw (×10^3^ M)	PDI
PK30	100	-	-	-	2.4	5.4	2.2
PK30_x_-FU_y_	35	65	-	65	2.1	5.4	2.6
PK30_x_-FU_y_-A2P_z_	28	55	17	72	1.8	5.0	2.7
PK30_x_-FU_y_-A2P_z_	27	47	26	73	2.0	5.4	2.7
PK30_x_-FU_y_-A2P_z_	28	36	36	72	1.9	5.3	2.7
PK30_x_-A2P_z_	28	-	72	72	1.7	4.1	2.4

## Supplementary Materials

The following are available online at https://www.mdpi.com/2073-4360/11/11/1885/s1. Table S1: Composition of polyketones functionalized with FU and A2P groups, Table S2: Experimental conditions of grafted polymers mixed with different wt.% of MWCNTs, Figure S1: Set up for electrical conductive easements of a bar (A) with clamps and (B) Infrared camera, Figure S2: DSC first cycle after thermal history erase of PK30 grafted with FU, A2P and their respective co-polymers, Figure S3: FT-IR of PK grafted with the ratios of FU and A2P Untried (non-cross-linked) and cross-linked with different wt.% of MWCNTs, Figure S4: DSC thermal cycles of PK-Fu-A2P series cross-linked with b-Ma and reinforced with different wt.% of MWCNTs, Figure S5: Three hot compression molded bars made with a grinded cross-linked sample (see powder in the middle of the picture) using 150 °C and 40 bar during 30 min., Figure S6 Dynamic mechanical analysis of bar samples made with PK30-FU60-A2P20, PK30-FU40-A2P40, PK30-FU20-A2P60 cross-linked with bismaleimide and reinforced with different wt.% of MWCNTs.
