# Supplementary material for: Electrically Self-Healing Thermoset MWCNTs Composites Based on Diels-Alder and Hydrogen Bonds
Source: Polymers (Basel). 2019 Nov 14;11(11):1885. doi: 10.3390/polym11111885 (PMC6918341; doi:10.3390/polym11111885)
Supplement: Supplementary file 1 [file polymers-11-01885-s001.pdf]

# Electrically self-healing thermoset MWCNTs nanocomposites based on Diels-Alder and hydrogen bonds

**Guilherme Macedo R. Lima [1], Felipe Orozco [1], Francesco Picchioni [1], Ignacio Moreno-Villoslada [2], Andrea Pucci [3], Ranjita K. Bose [1]* and Rodrigo Araya-Hermosilla [4,*]**

1. Department of Chemical Product Engineering, ENTEG, University of Groningen, Nijenborgh 4, 9747AG Groningen, The Netherlands; g.de.macedo.rooweder.lima@rug.nl (G.L.); f.orozco.gutierrez@rug.nl (F.O.); f.picchioni@rug.nl (F.P.)
2. Laboratorio de Polímeros, Instituto de Ciencias Químicas, Facultad de Ciencias, Universidad Austral de Chile, Valdivia 5090000, Chile; imorenovilloslada@uach.cl (I.M.-V.)
3. Department of Chemistry and Industrial Chemistry, University of Pisa, Via Moruzzi 13, 56124 Pisa, Italy; andrea.pucci@unipi.it (A.P.)
4. Programa Institucional de Fomento a la Investigación, Desarrollo e Innovación, Universidad Tecnológica Metropolitana, Ignacio Valdivieso 2409, P.O. Box 8940577, San Joaquín, Santiago 8940000, Chile

* Correspondence: rodrigo.araya@utem.cl (R.A.-H.); r.k.bose@rug.nl (R.B.);
Tel.: +56-2-27877911 (R.A.-H.); +31 50 36 34486 (R.B.)

*Table S1. Composition of polyketones functionalized with FU and A2P groups. The molar ratio between the reactants (Fu and A2P) in the feed is indicated by numbers code representing percentages that aims at a maximal 80% of conversion.*

| *Sample* | *PK30 (g)* | *Fu (g)* | *A2P(g)* | *Tot (g)* |
|---|---|---|---|---|
| *PK30-FU80* | *30,0* | *17,71* | *0,00* | *47,71* |
| *PK30-FU60-A2P20* | *30,0* | *13,28* | *3,38* | *46,66* |
| *PK30-FU40-A2P40* | *30,0* | *8,86* | *6,76* | *45,62* |
| *PK30-FU20-A2P60* | *30,0* | *4,43* | *10,14* | *44,57* |
| *PK30-A2P80* | *30,0* | *0,00* | *13,70* | *43,70* |

The PK30 was preheated to the liquid state at the employed reaction temperature (110 °C). Then FU and A2P were added dropwise to the reactor in the first 30 min in sequence. The stirring speed was set at a constant value of 600 rpm, and the employed reaction time was 4 h from the moment that the last drop was added. After the reaction, the color of the reaction mixtures changed from light yellow to a range of brown, depending on the ratio of FU/A2P. The resulting products were then allowed to cool down to room temperature before being dissolved with chloroform to facilitate the extraction from the glassware. The resulting polymer solutions were poured in a Teflon plate and let rest for 24h inside the fume hood, in sequence the plate was transferred to a vacuum oven at 50 ºC for 24 h to remove the solvent. The resulting polymers were frozen with liquid nitrogen, ground to powder and washed using Milli-Q water by stirring it for 15 minutes. The process was repeated three times to remove any leftover amine compounds. After filtering, the materials were dried in a vacuum oven at 60 ºC for 48h to remove the remaining Milli-Q water. In order to avoid hydration, the samples were sealed in brown glass vials and stored at 6 ºC for further characterization.

The percentage of conversion of carbonyls ($C_{co}$) into pyrrole groups can be calculated as follows;

$$C_{co} = \frac{y}{y + x} \times 100\% \qquad (1)$$

Where x and y represent the moles of di-ketone and pyrrolic units after conversion, respectively y can be calculated as follows;

$$y = \frac{wt(N)}{A_m(N)} \qquad (2)$$

Where wt(N) represents the grams of nitrogen in the final product according to the elemental analysis and $A_m(N)$ is the atomic mass of nitrogen. X can be calculated as follows:

$$x = \frac{g_{prod} - y \times M_w^y}{M_w^{pk}} \qquad (3)$$

Where $g_{prod}$ represents the grams of the product after conversion, $M_w^y$ the molecular weight of the pyrrolic unit and $M_w^{pk}$ the molecular weight of a 1,4 di-ketone unit (131,6 g/mol). The conversion efficiency $\eta$ is defined as the ratio between the carbonyl conversion $C_{co}$ and the targeted one according to the amount of polymer and amine compounds provided in the feed ($C_{co}^{feed}$)

$$\eta = \frac{C_{co}}{C_{co}^{feed}} \times 100\% \qquad (4)$$

The $C_{co}^{feed}$ is calculated as follows:

$$C_{co}^{feed} = \frac{Mol_{amine}}{Mol_{d-co}} \times 100\% \qquad (5)$$

With $Mol_{amine}$ representing the moles of amine compounds and $Mol_{d-co}$ the moles of di-carbonyl units in the feed.

*Table S2 Experimental conditions of grafted polymers mixed with different wt.% of MWCNTs*

| *Sample* | | *BisMa* | *MWCNT* | | *Total* |
|---|---|---|---|---|---|
| *Name* | *(g)* | *(g)* | *(%)* | *(g)* | *(g)* |
| *PK30-FU80* | *3,000* | *2,384* | *5,0%* | *0,270* | *5,654* |
| *PK30-FU60-A2P20* | *3,000* | *1,832* | *0,0%* | *0,000* | *4,832* |
| | *3,000* | *1,832* | *0,1%* | *0,005* | *4,837* |
| | *3,000* | *1,832* | *0,5%* | *0,024* | *4,857* |

| | | | | | |
|---|---|---|---|---|---|
| | *3,000* | *1,832* | *1,5%* | *0,074* | *4,906* |
| | *3,000* | *1,832* | *2,5%* | *0,124* | *4,956* |
| | *3,000* | *1,832* | *5,0%* | *0,254* | *5,087* |
| *PK30-FU40-A2P40* | *3,000* | *1,253* | *0,0%* | *0,000* | *4,253* |
| | *3,000* | *1,253* | *0,1%* | *0,004* | *4,257* |
| | *3,000* | *1,253* | *0,5%* | *0,021* | *4,274* |
| | *3,000* | *1,253* | *1,5%* | *0,065* | *4,318* |
| | *3,000* | *1,253* | *2,5%* | *0,109* | *4,362* |
| | *3,000* | *1,253* | *5,0%* | *0,224* | *4,477* |
| *PK30-FU20-A2P60* | *3,000* | *0,643* | *0,0%* | *0,000* | *3,643* |
| | *3,000* | *0,643* | *0,1%* | *0,004* | *3,647* |
| | *3,000* | *0,643* | *0,5%* | *0,018* | *3,661* |
| | *3,000* | *0,643* | *1,5%* | *0,055* | *3,698* |
| | *3,000* | *0,643* | *2,5%* | *0,093* | *3,736* |
| | *3,000* | *0,643* | *5,0%* | *0,192* | *3,835* |
| *PK30-A2P80* | *2.850* | *0,000* | *5,0%* | *0,150* | *3,000* |

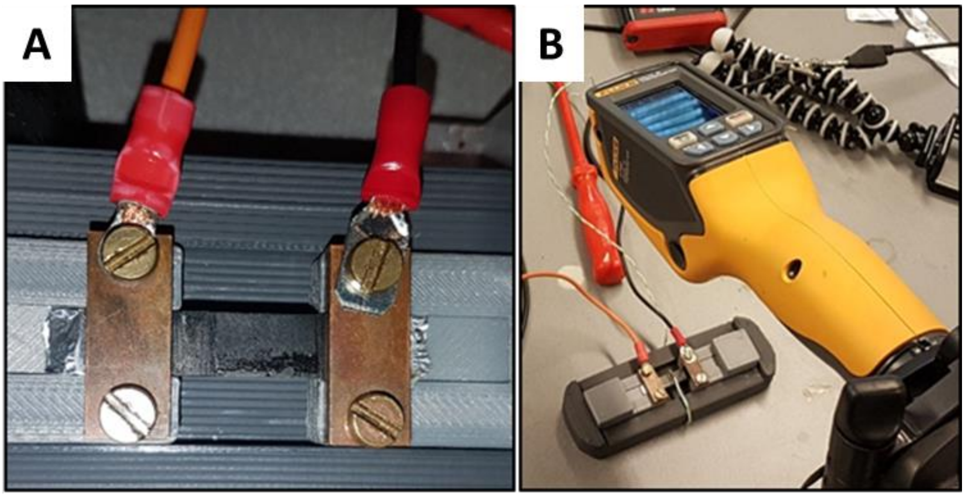


*Figure S1 Set up for electrical conductive easements of a bar (A) with clamps and (B) Infrared camera.*

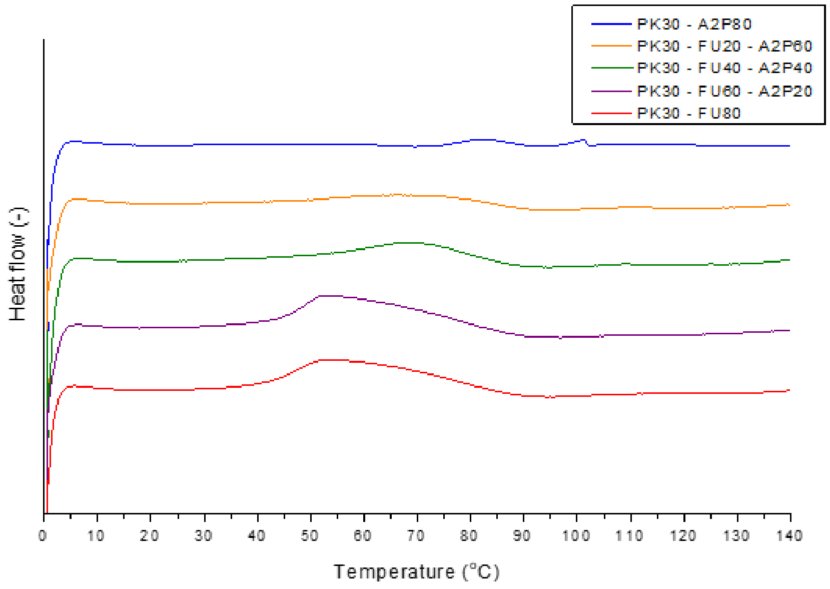


*Figure S2 DSC first cycle after thermal history erase of PK30 grafted with FU, A2P and their respective co-polymers.*

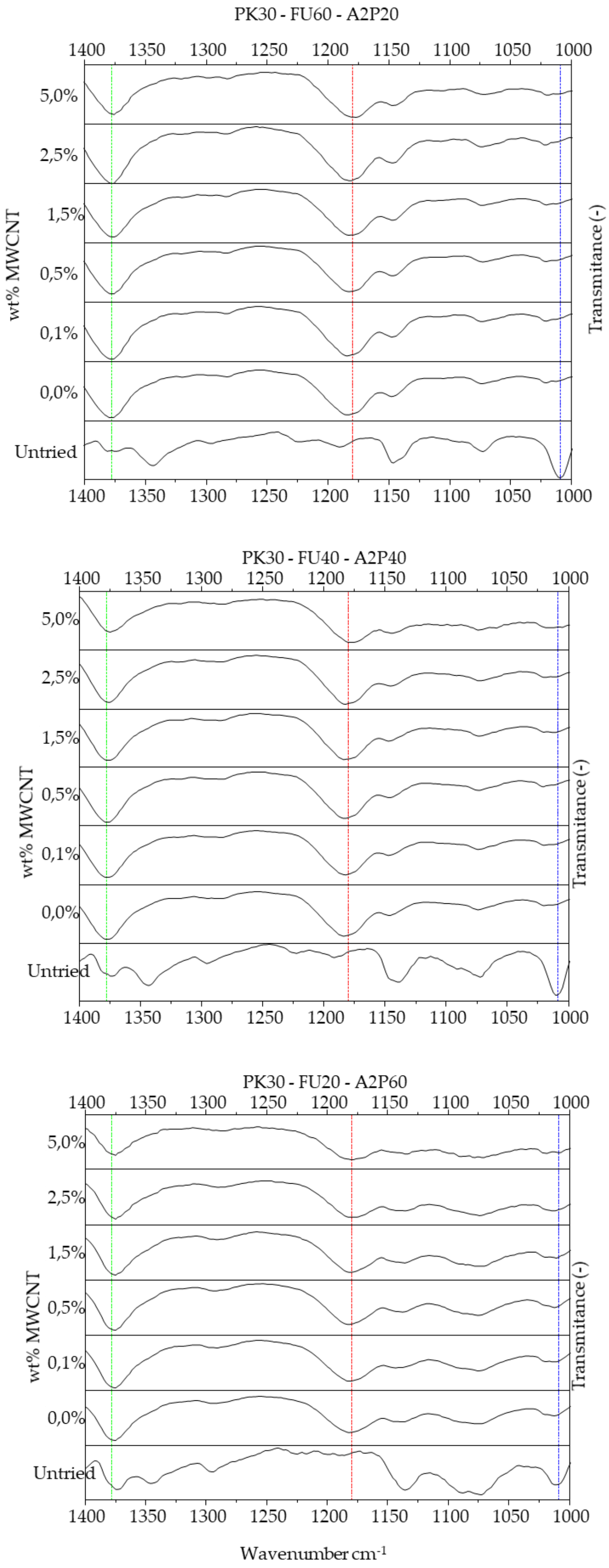


*Figure S3 FT-IR of PK grafted with the ratios of FU and A2P Untried (non-cross-linked) and cross-linked with different wt.% of MWCNTs.*

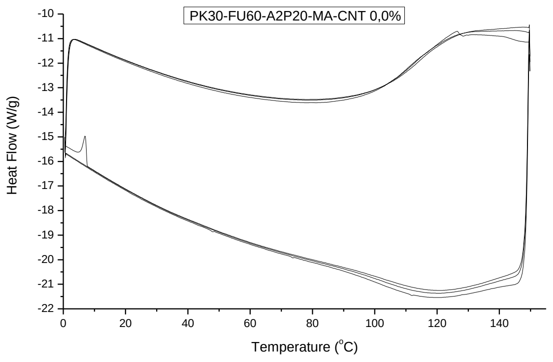
PK30-FU60-A2P20-MA-CNT 0,0%
Heat Flow (W/g)
Temperature (°C)

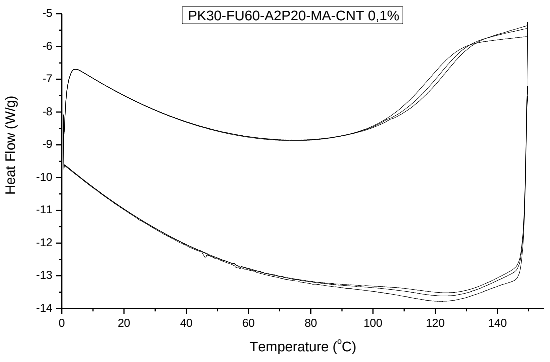
PK30-FU60-A2P20-MA-CNT 0,1%
Heat Flow (W/g)
Temperature (°C)

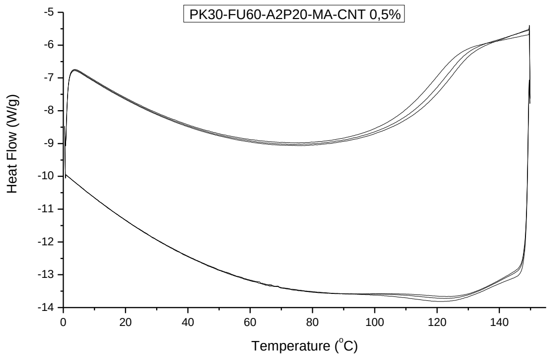
PK30-FU60-A2P20-MA-CNT 0,5%
Heat Flow (W/g)
Temperature (°C)

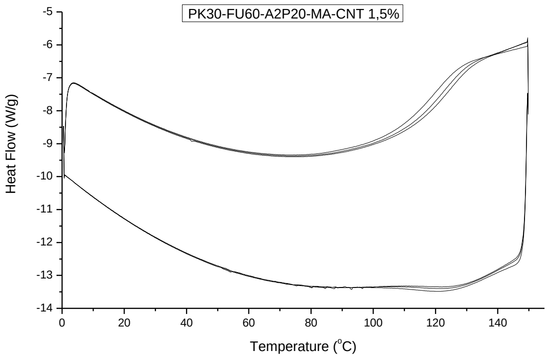
PK30-FU60-A2P20-MA-CNT 1,5%
Heat Flow (W/g)
Temperature (°C)

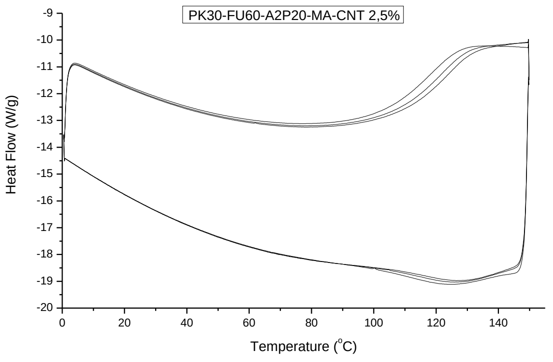
PK30-FU60-A2P20-MA-CNT 2,5%
Heat Flow (W/g)
Temperature (°C)

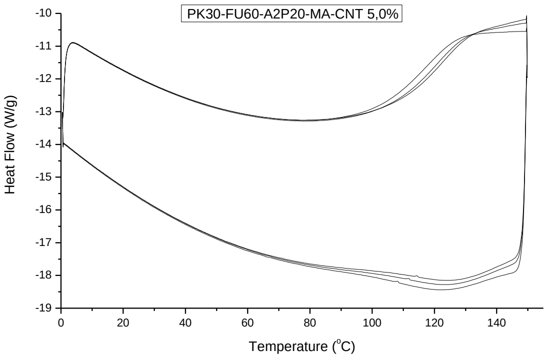
PK30-FU60-A2P20-MA-CNT 5,0%
Heat Flow (W/g)
Temperature (°C)

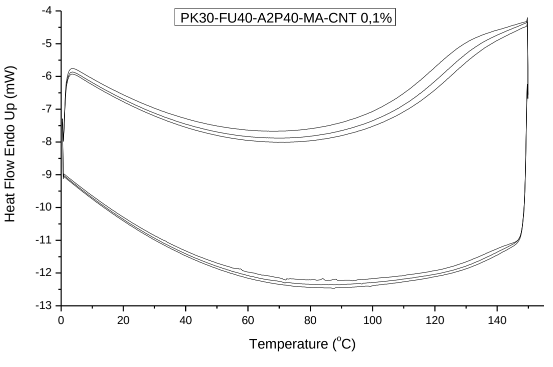
PK30-FU40-A2P40-MA-CNT 0,1%
Heat Flow Endo Up (mW)
Temperature (°C)

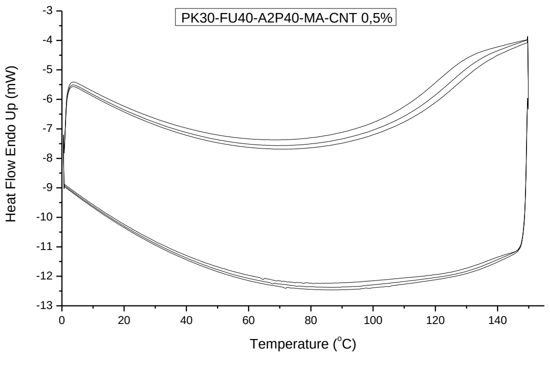
PK30-FU40-A2P40-MA-CNT 0,5%
Heat Flow Endo Up (mW)
Temperature (°C)

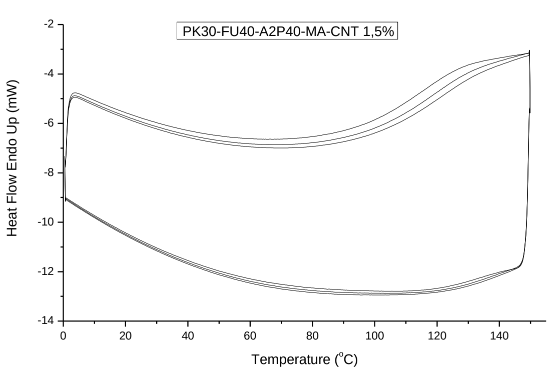
PK30-FU40-A2P40-MA-CNT 1,5%
Heat Flow Endo Up (mW)
Temperature (°C)

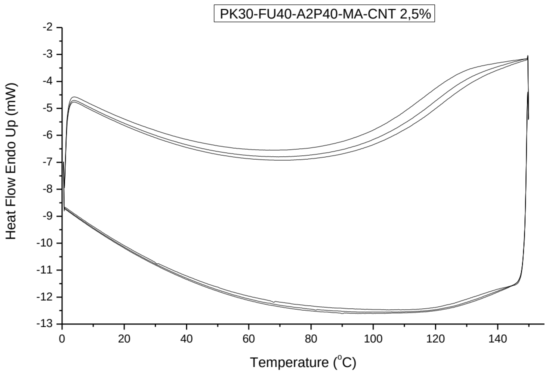
PK30-FU40-A2P40-MA-CNT 2,5%
Heat Flow Endo Up (mW)
Temperature (°C)

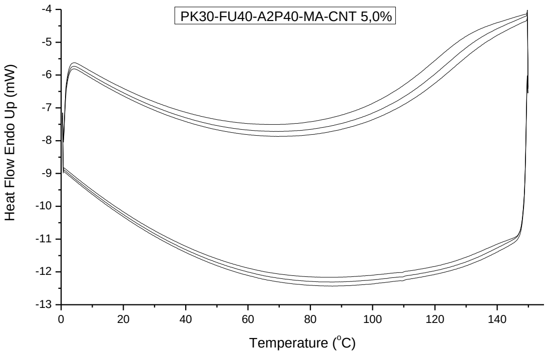
PK30-FU40-A2P40-MA-CNT 5,0%
Heat Flow Endo Up (mW)
Temperature (°C)

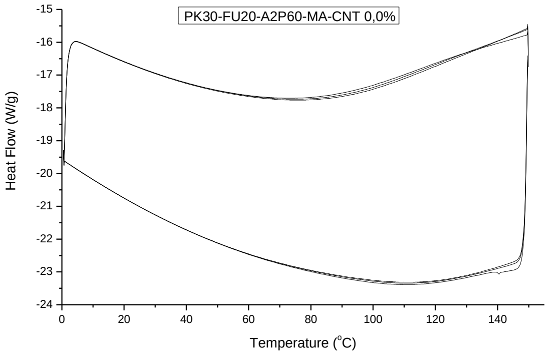
PK30-FU20-A2P60-MA-CNT 0,0%
Heat Flow (W/g)
Temperature (°C)

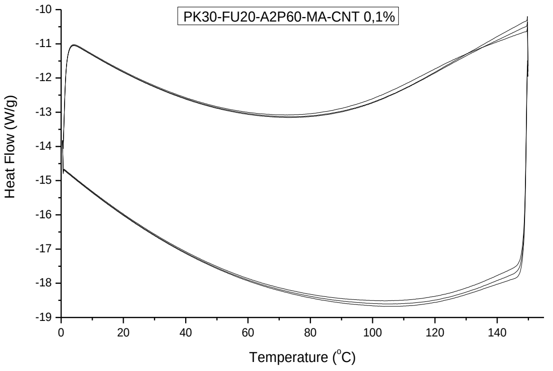
PK30-FU20-A2P60-MA-CNT 0,1%
Heat Flow (W/g)
Temperature (°C)

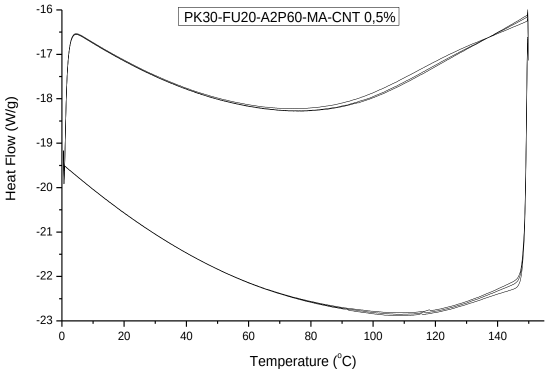
PK30-FU20-A2P60-MA-CNT 0,5%
Heat Flow (W/g)
Temperature (°C)

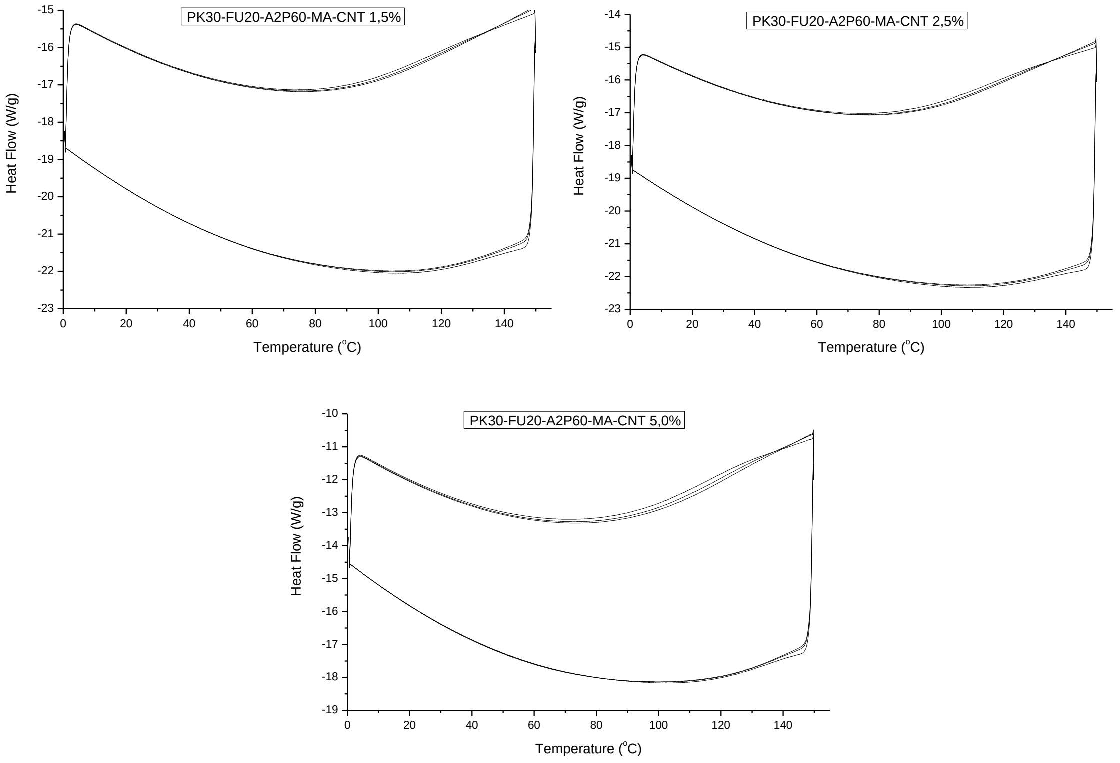


*Figure S4 DSC thermal cycles of PK-Fu-A2P series cross-linked with b-Ma and reinforced with different wt.% of MWCNTs.*

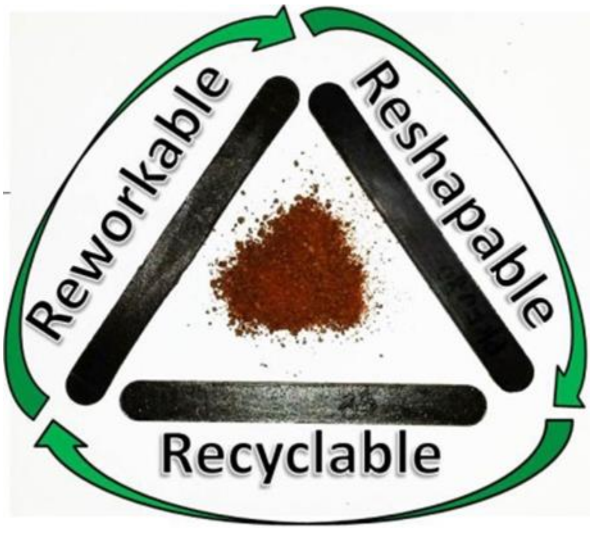


*Figure S5 Three hot compression moulded bars made with a grinded cross-linked sample (see powder in the middle of the picture) using 150 ℃ and 40 bar during 30 min.*

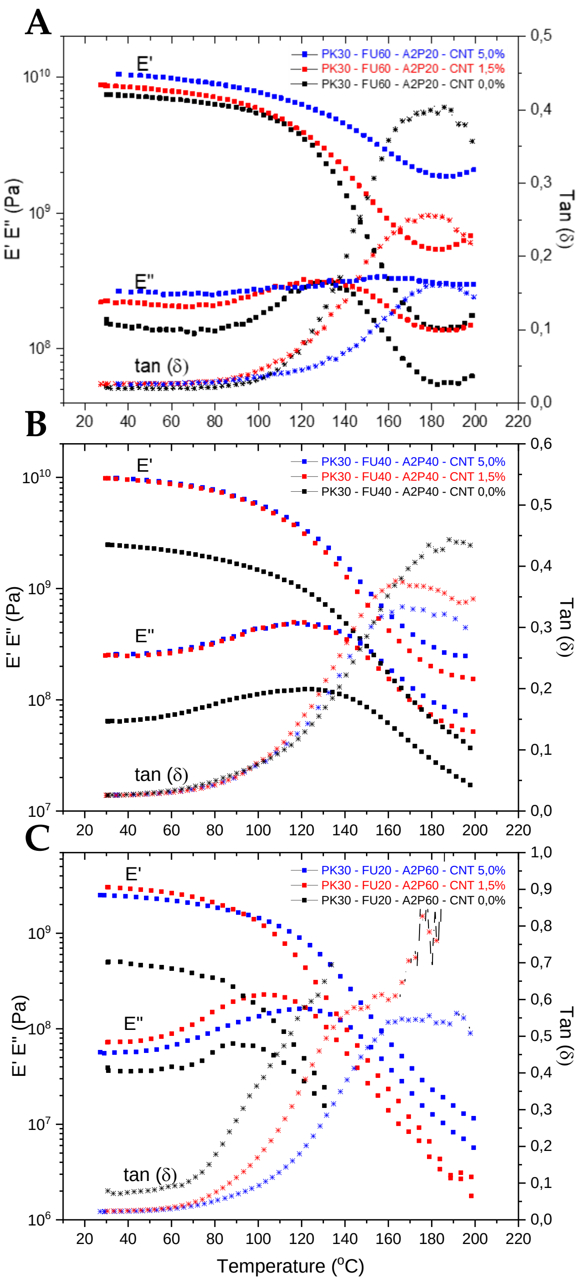


*Figure S6 Dynamic mechanical analysis of bar samples made with PK30-FU60-A2P20, PK30-FU40-A2P40, PK30-FU20-A2P60 cross-linked with bismaleimide and reinforced with different wt.% of MWCNTs.*
